# Anthropometric, Familial- and Lifestyle-Related Characteristics of School Children Skipping Breakfast in Jeddah, Saudi Arabia

**DOI:** 10.3390/nu12123668

**Published:** 2020-11-29

**Authors:** Hazzaa M. Al-Hazzaa, Amani A. Al-Rasheedi, Rayan A. Alsulaimani, Laura Jabri

**Affiliations:** 1 Lifestyle and Health Research Center, Health Sciences Research Center, Princess Nourah bint Abdulrahman University, Riyadh 11671, Saudi Arabia; 2 Food and Nutrition Department, Faculty of Human Sciences and Design, King Abdul Aziz University, Jeddah 42751, Saudi Arabia; aalrasheedi@kau.edu.sa; 3 Department of Pharmacology, Faculty of Medicine, King Abdul Aziz University, Jeddah 42751, Saudi Arabia; raalsulaimani@kau.edu.sa; 4 American International School of Jeddah, Jeddah 21352, Saudi Arabia; laurajabri@gmail.com

**Keywords:** breakfast intake, children, lifestyle behaviors, obesity, sociodemographic factors

## Abstract

Breakfast is a vital meal that provides children with important nutrients and energy. This study examined the anthropometric, familial- and lifestyle-related characteristics of school children skipping breakfast. A total of 1149 children (boys: 45.5%), 6 to 12 years old (mean and SD: 9.3 ± 1.7 years), were randomly selected from elementary schools in Jeddah. Weight and height were measured. Breakfast eating frequency, socio-demographics, and lifestyle behaviors were assessed using a specifically designed self-report questionnaire reported by the parents. Nearly 80% of the children skipped daily breakfast at home with no significant age or gender differences. The most common reasons for skipping breakfast at home included not feeling hungry and waking up late for school. Fried egg sandwiches and breakfast cereals were most frequently consumed for breakfast. Strong parental support for breakfast as the main daily meal was significantly associated with daily breakfast intake. Logistic regression analyses, adjusted for age, gender, and socio-demographics, revealed that paternal education (aOR = 1.212, 95% CI = 1.020–1.440, *p* = 0.029), maternal education (aOR = 1.212, 95% CI = 1.003–1.464, *p*=0.046), insufficient sleep (aOR = 0.735, 95% CI = 0.567–0.951, *p* = 0.019), and BMI <25 kg/m^2^ (aOR = 1.333, 95% CI = 1.015–1.752, *p* = 0.039) were significantly associated with breakfast intake. The findings have implications for children’s health and school performance. Concerted effort is required to promote breakfast consumption among Saudi children.

## 1. Introduction

Childhood obesity continues to be a global public health concern, along with its associated rise in cardiometabolic complications [1]. Meta-analysis research and a systematic review revealed that childhood overweight or obesity tracks well into adulthood, with 55% of obese children becoming obese adolescents and 80% of obese adolescents remaining obese in adulthood [2]. Worldwide, the prevalence of overweight and obesity among school-aged children and adolescents has risen enormously over the past decades [3]. In Saudi Arabia, a recent study conducted in Riyadh indicated that the prevalence of overweight plus obesity among children 6–8 years old and 9–11 years old were 24.6% and 30.9%, respectively [4]. Well-recognized major modifiable determinants of childhood obesity include diet and physical activity [5]. Although genetic factors may influence predisposition to obesity, a healthy—as opposed to unhealthy—lifestyle was reported to substantially lower the risk of obesity by 85% among children at high polygenic risk [6].

Regular breakfast intake in children has been shown to be associated with healthy body weight [7]. Numerous studies have indicated that skipping breakfast predisposes children and adolescents to obesity [8,9,10,11]. In one North American study, adolescents who consumed breakfast more often had a lower body mass index (BMI) than those who skipped breakfast [8]. In prospective analyses, frequency of breakfast intake among children from the USA was inversely associated with BMI in a dose-response manner [12]. Further, in longitudinal research from Japan, it was shown that skipping breakfast in early childhood increased overweight/obesity in later childhood [13]. In another longitudinal study involving Croatian adolescents, participants who consumed breakfast had significantly lower body fat percentages compared to those who skipped breakfast [14]. Local studies regarding breakfast and overweight or obesity status among Saudi children showed conflicting results [15,16]. While one study found that the majority of the underweight (94%) and obese children (89%) reported skipping breakfast [15], another recent study found no association between obesity level and breakfast consumption [16]. Different methods of sampling and calculating obesity status may have contributed for the differences in their results. Nevertheless, it is well recognized that the association between breakfast intake and obesity is confounded by many factors, including socioeconomic factors, home environment, circadian rhythms, and a variety of lifestyle behaviors such as eating fast food, physical activity and sedentary lifestyle [17]. 

Breakfast is a vital meal that provides children with important nutrients and energy. Consuming breakfast can improve cognitive learning and academic performance among children and adolescents [18,19,20]. In addition, children who skip breakfast have less healthful diets than children who regularly consume breakfast. Skipping breakfast was shown to disturb the adequacy of nutrient intake in a recent multicenter European study involving a large number of adolescents from ten cities [21]. It was also found that skipping breakfast leads to poorer diet quality among children and adolescents [22]. In addition, among Lebanese adolescents, skipping breakfast was associated with lower adherence to the Mediterranean diet [23]. 

Although regular breakfast consumption can have a multitude of positive health benefits, Saudi children were found to be more likely to skip breakfast than any other meal. A recent study on daily breakfast intake among Saudi children in Riyadh indicated that skipping an at-home breakfast has reached nearly 80% [16]. Skipping breakfast was found to be associated with insufficient sleep duration among Saudi children [24]. Furthermore, unhealthy lifestyle behaviors were shown to be negatively associated with daily breakfast behaviors in the Health Behaviour in School-aged Children study [25]. The high prevalence rate for skipping breakfast at home reported by children and adolescents in Saudi Arabia is very alarming and deserves further investigation [15,16,26,27,28]. Understanding the important familial, sociodemographic, and lifestyle determinants of breakfast consumption can also help in recognizing children at risk of skipping breakfast and may enhance our ability to plan and implement effective programs for preventing unhealthy breakfast intake behaviors in children. Therefore, in the present study, we report on the anthropometric, familial- and lifestyle-related characteristics among primary school children living in Jeddah, Saudi Arabia, relative to breakfast intake frequency and describe breakfast intake preferences by Saudi children relative to gender.

## 2. Materials and Methods

### 2.1. Study Design and Sample Selection

This is a cross-sectional study that was conducted in Jeddah during the 2019 school year. Jeddah, the second most populated city in Saudi Arabia, has a multiethnic population with over three million inhabitants. All Saudi children enrolled in boys’ and girls’ elementary schools from grades 1–6 during the study period were eligible for inclusion in the study. The exception was if the child had a medical condition related to eating disorders. In Saudi Arabia, schooling in grades 1–12 is mandatory and offered for free in public schools. The sample size was calculated with the assumption that the population proportion would yield the maximum possible sample size required (proportion = 0.50), with a confidence level of 95% and a margin of error of 4%. An additional 20% of participants were added to account for non-responders, or missing data. The total sample size for each gender was calculated to be 480 children, with 960 boys and girls in total. 

**Figure 1:**
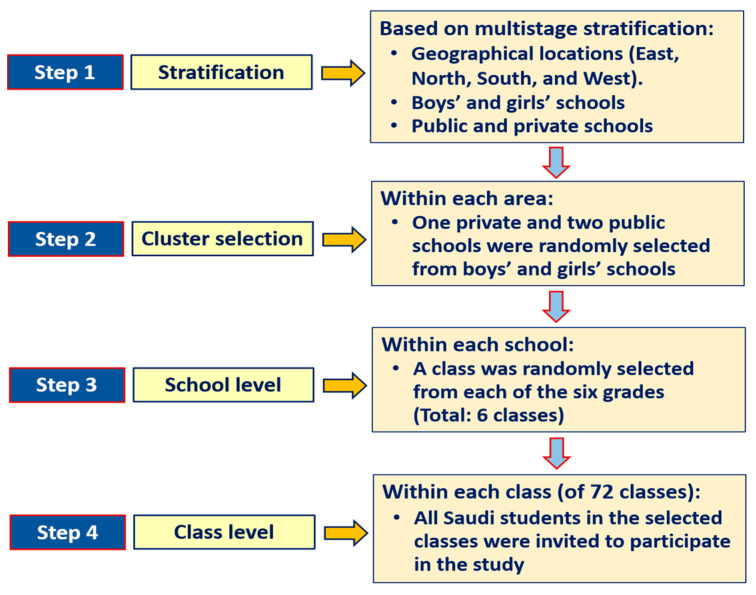
The protocol used for participants’ selection.

A representative random sample was selected from schools using a multistage stratified cluster sampling technique. Stratification was based on boys’ and girls’ schools (boys’ and girls’ schools are segregated in Saudi Arabia), public and private schools, as well as on major geographical locations (east, west, north, and south). Children were drawn from elementary schools relative to the actual number of students in public and private schools and geographical location in Jeddah. Within each area, one private and two public schools were randomly selected. Then, within each school, a class was randomly selected from each of the six grades. The final number of classes selected from all six grades was 72. All Saudi students in the designated classes were then invited to participate in the study. Figure 1 illustrates the protocol that was used for the students’ selection. Normally, there are about 25 Saudi students in each class in public schools and nearly 15 Saudi students in each class in private schools. Ethical approval was obtained from the Institutional Review Board (IRB) at Princess Nourah bint Abdulrahman University, Riyadh (IRB Log Number: 19-0014). The research procedures were conducted in accordance with the principles expressed in the Declaration of Helsinki. Written informed consent was obtained from all participating parents. Approval for conducting this research in schools was secured from the Jeddah directorate of schools, Ministry of Education, and the principals of the selected schools.

### 2.2. Anthropometric Measurement

Body weight was measured to the nearest 100 g using calibrated portable medical scales (Seca 869, Birmingham, UK). All measurements were taken with minimal clothing and without shoes by trained researchers. Height was measured to the nearest 0.1 cm using a measuring rod calibrated to the nearest centimeter while the subject was in a full standing position without shoes. Body mass index (BMI) was computed as the ratio of weight in kilograms divided by the squared height in meters. The extended International Obesity Task Force (IOTF) age- and sex-specific BMI cutoff reference standards were used to classify underweight, normal weight, and overweight or obesity relative to the child’s age [29].

### 2.3. Assessment of Breakfast Eating Habits

Breakfast eating habits and food preferences were assessed using a specifically designed self-report questionnaire that was filled out by the children’s parents [16]. With clear instructions, parents were asked to complete the questionnaire forms based on the child’s typical (habitual) breakfast habits. In addition, the questionnaire form included information on demographic and socioeconomic status. Additional questions were related to breakfast choices and behaviors as well as how satisfied the parents were with their child’s breakfast choices (three Likert scale; satisfied, somewhat satisfied, or not satisfied) and the level of importance of breakfast intake as a meal for their child (very important, somewhat important, or not important). A variety of common breakfast choices were provided in the questionnaire, including cheese, eggs, ready-to-eat cereals, pizza, peanut butter or hummus sandwiches, potatoes, sausage, cookies, and muffins. The questionnaire was previously developed and content validated and reviewed and agreed upon by three experts in the field of nutrition and dietary habits [16]. The actual questionnaire can be found as a supplementary file in a previous publication [16].

### 2.4. Assessment of Screen Time, Sleep, and Physical Activity

Assessment of screen time, sleep, and physical activity was part of the breakfast intake questionnaire. Questions related to screen time included items intended to determine information from the parents about the typical amount of daily screen time the child spent, including time spent watching TV, playing non-active video games, and using the computer and internet for recreational purposes. Parents were asked to provide the average usual hours spent during weekdays and weekends. For classifying screen time cutoff hours, we used the American Academy of Pediatrics guidelines and the Canadian 24-Hour Movement Guidelines for Children and Youth (ages 5–17 years) which call for a maximum of two hours per day of screen time [30,31].

Nocturnal sleep duration on weekdays (school days) and weekends was assessed using questions embedded within the questionnaire. Parents were asked how many hours their children usually sleep at night on weekdays and weekends. We defined insufficient sleep (short sleepers) as sleeping less than nine hours per night, according to the definition of the National Sleep Foundation for school-age children 6 to 13 years old [32].

Physical activity was assessed using the total daily time spent by the child on all types of physical activities, including sports, during which the child’s breathing was considerably increased. The sufficient physical activity level was based on 60 min or more of daily physical activity [33]. Accordingly, physical activity was classified as low or high activity based on a cutoff value of 420 min per week or the recommended daily time for children and adolescents [31,33].

### 2.5. Statistical Analysis

Data were entered into an SPSS data file, checked, cleaned, and analyzed using the IBM-SPSS software, version 22 (Chicago, IL, USA). Descriptive statistics were obtained for all variables and reported as means and standard deviations or percentages. Differences between boys and girls in selected descriptive measurements were tested using MANCOVA tests while controlling for socioeconomic status. Bonferroni test was used for testing in-between subject differences. Chi-square tests of proportions were used to test differences in sociodemographic factors and dichotomized lifestyle behaviors. Multivariable analyses (MANCOVA) were used to test differences in selected variables (gender and frequency of breakfast intake (below 5 days per week versus 5 or more days per week)) while controlling for age and sociodemographic factors. Since physical activity in minutes per week is not normally distributed, we used log transformation when analyzing physical activity in the MANCOV test. Wilks’ Lambda tests as multivariable test was reported as well as tests of between subject’s effects (Bonferroni test) for breakfast intake, gender, and the interactions of breakfast intake with gender. Finally, logistic regression analysis of selected lifestyle behaviors was used to test differences in the frequency breakfast intake (high versus low intake) among Saudi children, while adjusted for age, gender, and sociodemographic factors. Adjusted odds ratios (aOR) and confidence intervals (95% CI) were reported. Alpha level was set at 0.05, and *p*-value less than alpha level was considered significant.

## 3. Results

Table 1 displays the descriptive characteristics of the participants. The total number of the sample was 1,149 children (523 boys and 626 girls) between the ages of 6 and 13 with a mean age (SD) of 9.2 (1.7) years. No significant (*p* = 0.365) difference in age was observed between boys and girls. There were no significant differences between boys and girls in body weight (*p* = 0.246), height (*p* = 0.608), or BMI value (*p* = 0.055). Breakfast intake averaged 3.76 ± 2.3 days per week, with no significant (*p* = 0.778) differences between boys and girls. In addition, there were no significant differences between boys and girls in breakfast intake on weekdays (*p* = 0.809) or weekends (*p* = 0.793). The findings also showed that the prevalence of children having daily breakfast at home was 20.4%. The most noted reasons for those children not regularly having breakfast at home included the child not feeling hungry (47.6%), the child waking up late and not having time for breakfast at home (in which case, he/she was given a sandwich to eat on the way to school or at school) (36.1%), and the child being given money to buy food from the school canteen (26.7%). Furthermore, BMI classifications showed no significant (*p* = 0.093) difference between boys and girls in the proportions of underweight, normal weight, and overweight or obesity. The prevalence of overweight plus obesity for the entire sample reached 28.7%, with no significant differences relative to gender. 

The proportions (%) of Saudi children who exceeded certain cutoff values for breakfast intake, overweight/obesity, and selected lifestyle behaviors are presented in Table 2. Nearly 80% of the children did not consume a daily breakfast at home. Almost 70% of the children spent more than two hours of daily screen time, with boys being exceedingly more sedentary than girls. About 66% of the sample did not sleep for the proper duration at night (9 or more hours per night). In addition, the majority (84.8%) of the children were considerably less active than the recommended daily time for physical activity, which amounts to one hour of daily physical activity, with girls having significantly (*p* < 0.001) less physical activity levels than boys. A very small proportion (less than 5%) of the children walked to school.

Table 3 presents the sociodemographic and lifestyle factors of the participants in relation to the breakfast intake category (daily versus non-daily intake). There were no significant differences between children having breakfast for 5 or more days per week compared to those having breakfast for less than 5 days per week in many of the sociodemographic variables, such as gender, school type, parent answering the questionnaire, size of the family living in the house, maternal age, or family income. However, daily breakfast intake appears to associate significantly with lower paternal age, and higher paternal education status. In addition, daily breakfast intake is associated with sleeping sufficiently. 

The types of breakfast choices (%) consumed at home by Saudi children relative to gender are shown in Table 4. Varied foods were consumed during breakfast by the Saudi children; however, fried egg sandwiches (48.1%), breakfast cereals (45.9%), and spread cheese sandwiches (41.3%) were the foods most frequently consumed for breakfast, followed by boiled egg sandwiches (27.7%), tuna sandwiches (25.8%), and Nutella sandwiches (25.0%). Healthy breakfast choices, such as falafel sandwiches, thyme pie, labneh pie, and chickpea sandwiches were consumed less frequently (4–7%). Breakfast choices that were consumed significantly more often (for 5 or more days per week compared to less than 5 days per week) included cereals (58.1%), boiled egg sandwiches (32.9%), labneh sandwiches (21.4%), peanut butter sandwiches (12.4%), and solid cheese sandwiches (11.1%). 

Table 5 displays breakfast intake preferences by Saudi children relative to gender. Two thirds of the sample preferred to eat breakfast cereals with sugar, whereas nearly 16% preferred cereals with fruit, without any significant difference between boys and girls. Moreover, a higher percentage of boys (60.6%) than girls (46.8%) consumed fresh fruit at breakfast. Apples (31.8%) and bananas (24.6%) were the most consumed fruits for breakfast. Full fat milk (44.6%), water (42.2%), chocolate milk (31.6%), fruit juice (29.4%), and tea with milk (27.2%) were the most common breakfast drinks consumed by the children. Girls showed significantly (*p* = 0.003) higher chocolate milk intake at breakfast than boys. Full fat milk was associated with daily breakfast intake, while chocolate milk was associated with non-breakfast consumption. In addition, there was no association between fruit intake and daily breakfast consumption (not shown in the table). 

As shown in Table 6, mothers appear to prepare breakfast nearly 78% of the time, followed by domestic helpers (7.3%). Less than 2% of the children have lactose intolerance. In addition, about 46% of the parents were satisfied with their children’s breakfast intake. Parents placed high importance (93.0%) on the breakfast intake of their children, and this significantly impacted the children’s daily breakfast intake. Compared to lunch and dinner, 80.6% of parents regarded breakfast as the most important meal of the day for their children, and children with more frequent breakfast intake seemed to have parents with the strongest (*p* = 0.024) opinions on breakfast as the main meal of the day.

Table 7 shows the results of the multivariable analysis of selected anthropometric and lifestyle variables stratified by gender and frequency of breakfast intake (5 or more days per week compared to less than 5 days per week), while controlling for age and socio-demographic factors. There were significant differences in body weight, BMI, and sleep duration between children having more frequent breakfast intake versus less frequent breakfast intake, while there were significant differences relative to gender in screen time, sleep duration, and physical activity levels. In addition, the results of the logistic regression analyses of selected lifestyle behaviors relative to the frequency of breakfast intake, while adjusted for age, gender, and socio-demographic factors, are presented in Table 8. Father’s education status (aOR = 1.212, 95% CI = 1.020–1.440, *p* = 0.029), mother’s education (aOR = 1.212, 95% CI = 1.003–1.464, *p* = 0.046), insufficient sleep duration (aOR = 0.735, 95% CI = 0.567–0.951, *p* = 0.019), BMI status less than 25 kg/m^2^ (aOR = 1.333, 95% CI = 1.015–1.752, *p* = 0.039) were significantly associated higher frequency of breakfast intake. In another logistic regression model (not shown in a table), we included the type of breakfast choices as independent variables and the results showed that the following breakfast choices were associated with breakfast intake: breakfast cereal (aOR = 1.787, 95% CI = 1.384–2.308, p < 0.001), solid cheese sandwich (aOR = 1.684, 95% CI = 1.050–2.700, *p* = 0.031), and hot dog (aOR = 0.473, 95% CI = 0.259–0.866, *p* = 0.015). 

## 4. Discussion

The present study examined the anthropometric, familial- and lifestyle-related characteristics of Saudi primary school children relative to breakfast intake frequency in Jeddah, Saudi Arabia. The main findings indicated that nearly 80% of the children in this study skipped daily breakfast at home with no significant gender differences. The most common reasons for not having breakfast at home include not being hungry and waking up late for school. In addition, daily breakfast intake appears to associate significantly with the parental and maternal education status, getting sufficient nocturnal sleep, and having normal weight. Fried egg sandwiches, breakfast cereals, and spread cheese sandwiches were the foods most frequently consumed for breakfast. Further, strong parental support of breakfast as the main meal of the day was significantly associated with the child’s daily breakfast intake. Finally, logistic regression analysis adjusted for age, gender, and sociodemographic factors indicated that the parental and maternal education status, sufficient sleep duration, and having a BMI value less than 25 kg/m^2^ were significantly associated with more frequent child’s breakfast intake. 

The prevalence of children having daily breakfast at home in the present study was 20.4%, without significant differences relative to age or gender. These findings corroborated recent findings on breakfast consumption (20.7%) reported for primary school children in another major city of the country, Riyadh [16]. However, other earlier studies showed varied breakfast skipping rates. For instance, a small (*n* = 120) study conducted in Riyadh reported that 40.8% of Saudi schoolgirls aged 9 to 13.9 years consumed a daily breakfast and that skipping breakfast increased with advancing age and among children with obesity compared to those without obesity [28]. Among primary students in Abha, in the southern area of Saudi Arabia, breakfast intake was reported as a regular meal for 72% of the children [34]. Moreover, the prevalence of skipping breakfast among male school children from the northern region of Saudi Arabia was reported to be 33.5% [35]. However, in a more recent cross-sectional study (*n* = 384) involving female primary school students from Riyadh, only 2% of the school girls consumed breakfast 5 to 7 days a week [15]. In the current study, more than 40% of our sample consumed breakfast for 5 or more days per week. The differences in the prevalence of breakfast consumption or breakfast skipping rate among Saudi children reported in the above cited studies may generally reflect different questionnaire designs, a small sample size in some studies, or unintended recruitment bias of selected children in other studies.

Elsewhere, the findings from the Health Behaviour in School-aged Children study (HBSC), which included participants from 41 countries, indicated that daily breakfast intake varied from 33% for Greek girls to 75% for Portuguese boys and that low breakfast intake was found in girls and children living in single parent households in many of the participating countries [25]. Furthermore, a lower rate of skipping breakfast was reported among Australian children between the ages of 2 and 17, and skipping breakfast was associated with being female, older, underweight or overweight/obese, and having lower physical activity, inadequate sleep, lower household income, socio-economical disadvantages, and living in a single-parent home [36]. In a study conducted in eight European countries, daily breakfast consumption reported by children varied from 56% in Slovenia to 92% in Spain on weekdays and from 79% in Greece to 93% in Norway on weekends [37]. Among a large group of Greek school children aged 7 to 18 years, 22.4% of boys and 23.1% of girls skipped breakfast [38]. In Bangkok, Thailand, 79% of elementary school children consumed breakfast every day [39].

The findings from the current study showed that logistic regression analysis, while adjusted for age, gender, and sociodemographic factors, indicated that children with BMI value lower than 25 kg/m^2^ was significantly associated with more frequent breakfast intake. Local studies regarding breakfast and overweight or obesity status showed conflicting results. While one study found that the majority of the underweight (94%) and obese children (89%) reported skipping breakfast [15], another study, from the northern region of the country, stated that regular consumption of breakfast at home resulted in a normal BMI and a reduced likelihood of being underweight in male school children [35]. Further, a significant negative association between breakfast intake and BMI was observed in a large group of Saudi adolescents sampled from three major cities in the country [40]. However, a recent study involving male and female primary school students from Riyadh did not find any significant association between obesity levels and skipping breakfast [16]. Elsewhere, numerous studies from various countries have indicated that regular breakfast consumption is repeatedly associated with healthy body weight [7,8,13,14]. In addition, a systematic review conducted on the dietary correlates of obesity among children in the Middle East showed that several dietary behaviors, including skipping breakfast, were associated with obesity [41]. 

It is well recognized that the association between breakfast intake and obesity is confounded by many factors. Therefore, not all studies report a relationship between breakfast consumption and obesity. For instance, a recent cross-sectional study observed no association between breakfast consumption and children being overweight/obese [42]. Additionally, in a meta-analysis of observational studies, the cross-sectional studies showed that the risk of obesity in children and adolescents skipping breakfast was 43% greater than those who consumed breakfast regularly, while no significant association was found in cohort studies [43]. However, the number of cohort studies included in the meta-analysis was very small [43]. A recent review summarizing the relationship of skipping breakfast with body weight and metabolic outcomes in the child and adolescent population concluded that skipping breakfast was associated with overweight or obesity in 94.7% of the subjects [17]. Nonetheless, there were some heterogeneities in the definitions of overweight or obesity, skipping breakfast, and nutrient assessment, and confounding factors were infrequently reported [17]. This association between overweight/obesity, however, does not establish a causal relationship between them [44].

In the present study, a variety of foods were consumed by the Saudi children. Food choices made by children can be influenced by multiple factors, including family setting, friends, socioeconomic status, health considerations, food availability, color and taste, religious practices, and consumer trends. The findings showed that fried egg sandwiches, followed by breakfast cereals and spread cheese sandwiches were the foods most frequently consumed for breakfast, and those who consumed more cereals, labneh sandwiches, peanut butter sandwiches, and solid cheese sandwiches for breakfast were more likely to consume a daily breakfast. Some of the previous choices for breakfast were considered healthy choices, containing proteins and plenty of nutrients. A large body of epidemiological research has indicated that higher intake of ready-to-eat cereals (RTEC) by children is more likely to meet their recommended daily nutrient intake [45,46,47]. Indeed, RTEC consumption has been found to contribute to adequate key nutrient intake among children and adolescents in nationally representative cross-sectional data from the Canadian Community Health Survey (2015) [48]. The survey also showed that the nutrient density of the diet, as defined by the Nutrient-Rich Food Index, was significantly higher among RTEC consumers compared to non-consumers. In addition, RTEC consumption was not linked to overweight or obesity in this community study [48].

The study findings indicate that a higher percentage of boys (60.6%) than girls (46.8%) consumed fresh fruit at breakfast and that apples and bananas were the most consumed fruits with breakfast. A recent study from Riyadh, Saudi Arabia, reported contrasting findings to the ones found in the present study in relation to fruit consumption during breakfast, as girls consumed significantly more fresh fruit at breakfast than boys [16]. The present study showed that fruit intake in general was not associated with daily breakfast consumption. However, the Danish Health Behaviour in School-Aged Children Study (HBSC) reported that irregular breakfast consumption among adolescents was associated with a low frequency of fruit and vegetable consumption [49]. In addition, among Iranian school students aged 7 to 18 years, significant associations were found between skipping main meals, including breakfast, and low intake of fruits and vegetables [50].

Previous studies have shown that several factors can influence breakfast intake, including age, gender, family structure, and socio-economic status [12,19,37,51]. In the current study, there were no differences in breakfast intake due to age or gender. In agreement with the current findings, a recent study conducted on school children in Riyadh found similar results regarding breakfast consumption in relation to age or gender [16]. Findings from a Greek study showed that children of parents having more than 14 years of education were more likely to consume a daily breakfast [37]. Previous studies in various populations have reported a positive association between regular breakfast consumption and socioeconomic status [12,52,53]. In addition, there were a variety of familial-related factors that may influence breakfast consumption. Indeed, the family environment is considered an important influence on the dietary behaviors of children; therefore, family structure should be considered when designing programs to promote healthy breakfast habits [54]. In the current findings, mothers appeared to prepare breakfast most of the time, followed by domestic helpers. However, having domestic helpers prepare breakfast for the child appeared to associate with higher daily breakfast intake. A previous study conducted on Saudi children reported similar findings; mothers generally prepared breakfast at home, followed by domestic helpers. However, having breakfast prepared by a domestic helper was associated with non-daily breakfast consumption in that study [16].

The most prevalent reasons for not having regular breakfast at home in the present study included the child was not feeling hungry, the child waking up late with no time to have breakfast at home, and the child being given money to buy food from the school canteen. In another local study conducted on schoolgirls from Riyadh, the main reasons for not eating breakfast included not feeling hungry, not having enough time, or not having the preferred food choices available to them [15]. Needless to say, parents play an important role in children’s eating behaviors through their attitudes and eating habits. In the present study, despite placing high importance (93.0%) on their children’s breakfast intake, only 46% of the parents appeared satisfied with their children’s breakfast intake. Those parents who considered breakfast to be a very important meal for their children were more likely to have their children consume daily breakfast. In a local study involving Saudi children from the central region of the country, parents also placed very high importance on breakfast compared to lunch or dinner [16]. Elsewhere, similar findings were reported by parents of elementary school-aged children in Bangkok, Thailand [39].

Unhealthy lifestyle behaviors were exhibited by a good proportion of children in the present study. Almost 70% of the children spend more than two hours of screen time daily, with boys being exceedingly spending more time on screen viewing than girls. Moreover, the majority of the children are considerably less active than the recommended daily time for physical activity, which is one hour of daily physical activity, with girls having significantly less physical activity than boys. Daily breakfast intake among school children was reported to be associated with a lower proportion of TV viewing time of more than two hours per day [25]. More screen time (more than two hours per day) increased the odds of skipping breakfast by almost 80% among Greek school children [38]. However, a study involving younger children from New Zealand aged 5 to 14 years found no association between breakfast intake and physical activity [55]. Findings from a multinational, cross-sectional study in 12 countries with 6228 children aged 9 to 11 years indicated that frequent breakfast consumption in the morning was related to a higher proportion of time spent in moderate to vigorous physical activity and a lower proportion of screen time compared with rare breakfast intake [56]. In a previous study conducted on Saudi adolescents, it was found that healthy versus unhealthy lifestyle habits cluster, as healthful dietary habits (intake of breakfast, fruits, vegetables, and milk) associate mostly with higher levels of physical activity, whereas unhealthful dietary habits (consumption of sugar-sweetened drinks, fast foods, cakes/donuts, and energy drinks) were related mostly to screen time [57].

It is well documented that adequate nocturnal sleep duration is an important part of maintaining good health and a sense of wellbeing among children and adolescents [58]. Two thirds of the sample in the present study did not have sufficient sleep duration at night (9 or more hours per night); however, more frequent breakfast intake was positively associated with sufficient nocturnal sleep among the current sample. A recent study conducted on children in Riyadh indicated that insufficient sleep duration was significantly associated with skipping daily breakfast [24]. More frequent intakes of breakfast were also reported to be significantly associated with adequate sleep duration among Saudi adolescents [59]. Indeed, the combination of insufficient sleep and skipping breakfast may have adverse effects on children’s health. Numerous studies have reported a significant relationship between adequate sleep and daily breakfast intake. In a large group of Greek school children, insufficient sleep (<8 to 9 h per day) increased the odds of skipping breakfast by almost 80% [38]. Among a large number of Australian school children and adolescents, those who missed breakfast reported significantly poorer sleep [60]. Additionally, the proportion of Japanese adolescents who regularly consumed breakfast was significantly higher among those who slept for long durations [61]. In agreement with previously mentioned studies, adequate sleep among Taiwanese adolescents aged 13 to 18 years was related to eating a healthy diet, including having a daily breakfast [58]. 

### Strengths and Limitations

The strengths of the present study include using a large and representative sample of Saudi children from both public and private schools in Jeddah. The study’s analysis adjusted for important confounders, such as age, gender, and socioeconomic status. However, the present study has some limitations for consideration. First, the study has a cross-sectional design, which precludes us from inferring a causal relationship between breakfast intake and the selected variables. Second, the study did not exactly account for the cluster design effect. However, the size of the cluster was kept relatively small (nearly 16 students per class) and the sample size was further increased by 20% above the calculated one. Third, the breakfast intake and food choices were assessed using a questionnaire completed by the child’s parents. Questionnaires are generally subject to recall bias and social desirability effects. Recall bias occurs when participants do not remember previous events or experiences accurately; however, asking the parent about routine or frequent events, such as information on typical breakfast intake of their children can minimize the recall bias to a great extent. Self-reporting bias represents an important problem in the assessment of most observational study, such as cross-sectional research. However, the anonymity of the respondent in the present study was an assuring factor to the parent. Fourth, although a clear definition of breakfast and adequate instructions were given to the parents, breakfast meaning can be subjectively interpreted by the respondents, as habitual breakfast is likely to vary among participants [62,63]. Fifth, Jeddah is a large cosmopolitan city with people from all parts of the country; however, the breakfast choices of the children in this city may not exactly reflect those of children living in rural areas of Saudi Arabia. Sixth, apart from the type of breakfast consumed, no further information on the quality and quantity of breakfast was assessed, as these two measures could be important in determining its health effects. Seventh, due to the large number of participants, we used a subjective assessment of physical activity rather than a more objective measure, such as motion sensors, which may have influenced the accuracy of the physical activity assessment. Therefore, the absolute physical activity levels presented in the current study should be interpreted with caution. 

## 5. Conclusions

Skipping breakfast was found to be very prevalent among Saudi children, with no significant age or gender differences found. These findings corroborated earlier findings on breakfast consumption in other cities of the country. The most common reasons for not having breakfast at home included not being hungry and waking up late for school. Fried egg sandwiches, breakfast cereals, and spread cheese sandwiches were the foods most frequently consumed for breakfast. Strong parental support of breakfast as the main meal of the day was significantly associated with the more frequent breakfast intake in children. In addition, univariate analysis indicated that daily breakfast intake appears to associate significantly with the paternal and maternal education, getting sufficient nocturnal sleep, and having normal weight. However, logistic regression analysis adjusted for age, gender, and sociodemographic factors revealed that both paternal and maternal education, adequate sleep duration, and having BMI value lower than 25 kg/m^2^ were all significantly associated with the more frequent breakfast intake in children. 

The present findings have implications for children’s health and school learning performance and indicate a need for concerted effort to promote daily breakfast consumption among Saudi school children. In addition, the findings of the present study in Jeddah, along with results from a similar study in Riyadh, which both show a high prevalence of skipping breakfast among Saudi children, raise a public health challenge. Prevention of breakfast skipping, increasing regular physical activity and reducing insufficient sleep and screen time can contribute to favorable lifestyle and dietary behaviors, as well as better overall health of Saudi children. Therefore, school children could be targeted for interventions to improve breakfast intake habits and overall lifestyle behaviors. Further, it is recommended that a nutrition education training for parents to be explored, in order to encourage and improve breakfast consumption habits among their school-age children.

## Figures and Tables

**Table 1:** Descriptive characteristics of the participants relative to gender.

Variable	All (*N* = 1149)	Boys (*N* = 523)	Girls (*N* = 626)	*p*-Value *
Age (year)	9.3 ± 1.7	9.3 ± 1.6	9.4 ± 1.7	0.365
Body weight (kg)	32.8 ± 11.3	32.4 ± 11.1	33.2 ± 11.5	0.246
Body height (cm)	133.2 ± 11.4	133.3 ± 10.5	133.0 ± 12.1	0.608
Body mass index (kg/m^2^)	18.1 ± 3.9	17.8 ± 4.0	18.3 ± 3.9	0.055
Breakfast intake (days/week)	3.76 ± 2.3	3.75 ± 2.3	3.76 ± 2.2	0.778
BMI category (%)				0.093
Underweight	15.4	17.9	13.3	
Normal weight	55.9	54.7	56.9	
Overweight	19.4	17.5	21.1	
Obesity	9.3	10.0	8.8	
Overweight + obesity	28.7	27.5	29.8	

Data are means ± standard deviations or percentage. * MANCOVA tests while controlling for socioeconomic status (Wilks’ Lambda for gender = 0.017). Chi Squares tests for the differences in proportions (BMI category).

**Table 2:** The proportions (%) of Saudi children who exceeded certain cut-off values for breakfast intake, overweight/obesity, and selected lifestyle behaviors.

Variable	Criterion *	Proportion (%)			*p*-Value **
		All (*N* =1149)	Boys (*N* = 523)	Girls (*N* = 626)	
Breakfast intake at home	Non-daily intake	79.6	78.8	80.4	0.509
Overweight or obesity	BMI > cut-offs	28.7	27.5	29.8	0.378
Screen time	>2 h/day	69.5	77.8	62.6	<0.001
Nocturnal sleep duration	<9 h	65.8	67.5	64.4	0.267
Physical activity	<420 min/week	84.8	74.8	93.1	<0.001
Walking to school	Not walking to school	95.7	95.0	96.3	00.005

* Overweight or obesity cut-offs are based on IOTF cut-off values [29], screen time cut-offs are based on reference [30,31], sleep duration cut-offs are based on [32], and physical activity cut-offs are based on [33]; ** Tests for the differences in proportions between boys and girls.

**Table 3:** Socio-demographic and lifestyle factors of the participants relative to high versus low breakfast intake frequency.

Variable	<5 Days/Week (*N* = 685)	5 + Days/Week (*N* = 464)	*p*-Value *
Breakfast intake (day per week)	2.1 ± 1.1	6.3 ± 0.83	
Gender (%)			0.923
Boys	45.4	45.7	
Girls	54.6	54.3	
School type (%)			0.490
Public	76.4	74.6	
Private	23.6	25.4	
Parent answering the questionnaire (%)			0.099
Father	38.5	33.4	
Mother	58.1	64.2	
Someone else	3.4	2.4	
Number of children in the family (%)			0.019
1–2	14.2	18.3	
3–4	49.9	53.0	
5+	35.9	28.7	
Number of family members in the house (%)			0.272
1–3	45.7	50.0	
4–5	50.9	47.6	
6+	3.4	2.4	
Paternal age (%)			0.019
<30 years	0.7	0.4	
30-39 years	28.8	32.5	
40–49 years	47.2	51.3	
50–59 years	19.0	13.4	
60+ years	3.4	2.4	
Maternal age (%)			0.057
<30 years	8.2	9.1	
30–39 years	60.3	64.4	
40–49 years	27.2	24.8	
50–59 years	4.4	1.7	
60+ years	0.00	0.00	
Paternal education (%)			0.001
Intermediate or less	15.2	9.3	
High school	30.8	25.4	
University degree	46.1	54.3	
Post graduate degree	7.9	11.0	
Maternal education (%)			<0.001
Intermediate or less	14.0	6.9	
High school	30.2	28.2	
University degree	52.3	59.5	
Post graduate degree	3.5	5.4	
Family income (%) **			0.146
10,000 SR or less	45.8	42.5	
10,001–20,000 SR	37.8	41.2	
20,001–30,000 SR	11.2	13.4	
30,001 + SR	5.1	3.0	
Screen time			0.728
≤2 h/day	30.1	31.0	
>2 h/day	69.9	69.0	
Sleep duration			0.001
<9 h/night	69.5	60.3	
≥9 h/night	30.5	39.7	
Physical activity (%)			0.840
No physical activity	53.1	50.9	
Less than 30 min/day	20.6	21.6	
30 min to less than 60 min/day	11.8	11.2	
60 min/day	8.3	10.6	
More than 60 min/day	3.5	3.4	
Physical activity/inactivity (%)			0.372
Low active	85.5	83.6	
High active	14.5	16.4	
Means of travelling to school (%)			0.291
Walking	4.8	3.4	
Family or private car	86.9	89.9	
School bus	8.3	6.7	
BMI category			0.057
<25 kg/m^2^	69.1	74.3	
25 + kg/m^2^	30.9	25.7	

* Chi-Square tests for the differences in proportions between daily and non-daily breakfast intake; ** SR = Saudi Riyal = 3.75 U$.

**Table 4:** Types of breakfast (%) eaten at home by Saudi children in relation to relative to high versus low breakfast intake frequency (more than one choice was possible).

Variable	Breakfast Intake			*p*-Value *
	All (*N* = 1149)	<5 Days/Week (*N* = 685)	5 + Days/Week (*N* = 464)	
Fried egg sandwich	48.1	46.1	51.1	0.100
Breakfast cereals	45.9	39.0	56.0	<0.001
Spread cheese sandwich	41.3	39.3	44.4	0.083
Boiled egg sandwich	27.7	25.5	30.8	0.050
Tuna sandwich	25.8	23.9	28.4	0.087
Nutella sandwich	25.0	23.4	27.4	0.123
Croissant	20.3	21.3	18.8	0.289
Labneh sandwich **	16.5	14.2	20.0	0.008
Cheese pie (Fataer Jubin)	14.2	15.2	12.7	0.240
Pancake	13.3	13.9	12.5	0.503
Fava beans (Foul)	12.7	13.0	12.3	0.724
Peanut butter sandwich	8.3	6.7	10.6	0.020
Thyme sandwich	7.8	7.3	8.6	0.413
Cake or cookies	7.6	7.4	7.8	0.844
Pizza	7.6	7.3	8.0	0.671
Mortadella sandwich	7.4	8.5	5.8	0.092
Solid cheese sandwich	7.4	5.5	10.1	0.004
Oreo biscuit/other types of biscuit	7.2	6.7	8.0	0.419
Falafel sandwich	6.9	7.4	6.0	0.354
Jam sandwich	6.6	5.7	8.0	0.127
Thyme pie (Fataer Zatar)	6.4	6.6	6.0	0.715
Labneh pie (Fataer Labneh)	6.4	6.0	6.9	0.534
Hot dog	5.5	6.7	3.7	0.026
Yogurt-with or without fruits	4.6	4.4	5.0	0.647
Chickpeas (Hummus) sandwich	4.2	3.9	4.5	0.627
Other kinds of breakfast ***	12.4	9.9	16.2	00.002

* Chi Squares tests of the proportions for significant difference between frequency of breakfast intake; ** Soft, cream cheese made from strained yogurt, popular in Middle Eastern cuisine; *** Below 4% and include French fries, baked potato, donuts, hamburger, lentils, sweet Arabic pastry, or honey sandwich.

**Table 5:** Some breakfast intake preferences at home by Saudi children relative to gender (more than one choice was possible).

Variable	All (*N* = (1149)	Boys (*N* = (523)	Girls (*N* = (626)	*p*-Value *
Preference for cereal with/without added sugar (%)				0.194
With sugar	66.9	65.3	68.1	
Without sugar	33.1	34.7	31.9	
Preference for cereal with or without fruit (%)				0.700
With fruit	15.6	14.6	16.5	
Without fruit	84.4	85.4	83.5	
Percentage of fresh fruit eaten with breakfast (%)	53.1	60.6	46.8	<0.001
Types of fruits consumed most with breakfast (%):				
Apple	31.8	26.8	35.9	0.001
Banana	24.6	26.0	22.9	0.237
Grape	16.6	12.4	20.1	<0.001
Orange	15.9	14.5	17.1	0.237
Strawberries	8.6	3.8	12.6	<0.001
Pear	5.3	6.4	4.0	0.074
Mango	1.8	1.7	1.9	0.805
Kiwi	1.5	1.5	1.4	0.898
Watermelon	1.0	1.0	1.0	0.997
All others (less than 1% each and include dates, pineapple, figs and raisins)	1.1	0.3	2.0	<0.001
Drinks consumed with breakfast (%)	90.5	95.0	86.7	<0.001
Types of Drinks consumed with breakfast (%):				
Full fat milk	44.6	44.0	45.2	0.676
Water	42.2	39.2	44.7	0.095
Chocolate milk	31.6	27.2	35.3	0.003
Fruit juice	29.4	27.3	31.2	0.158
Tea with milk	27.2	28.7	25.9	0.279
Tea	11.6	13.8	9.7	0.034
Strawberry milk	9.7	6.7	12.3	0.001
Fruit drink	9.4	8.8	9.9	0.521
Low fat milk	8.2	9.4	7.2	0.179
Soft drink (soft beverage)	2.0	3.3	1.0	0.006
Butter milk	1.0	1.3	0.8	0.370
Coffee with milk	0.7	1.0	0.5	0.333
Other drinks	1.0	0.0	1.9	0.333

* Chi Squares tests of the proportions for significant difference between males and females.

**Table 6:** Factors related to breakfast intake and parent’s perception of child’s breakfast consumption relative to breakfast intake frequency.

Variable	Frequency of Breakfast Intake			*p*-Value *
	All (*N* = 1149)	<5 Days/Week (*N* = 685)	5 + Days/Week (*N* = 464)	
Who prepare the breakfast the most for the child? (%)				0.019
Mother	77.9	76.8	79.5	
Domestic helper	7.3	7.0	7.8	
The child himself/herself	4.4	5.8	2.4	
Father	3.6	3.5	3.7	
Nothing is prepared at home	3.0	3.8	1.9	
Sister/brother	2.2	1.5	3.2	
Brought ready from the market	1.6	1.6	1.5	
Does your child have lactose intolerance? (%)				0.266
Yes	1.8	2.2	1.3	
No	98.2	97.8	98.7	
Are you satisfied with the breakfast consumed by your child at home? (%)				0.001
Yes, satisfied	45.7	43.4	49.1	
Somewhat satisfied	46.7	46.7	46.8	
Not satisfied	7.6	9.9	4.1	
As a meal, how important for you is your child’s breakfast? (%)				0.069
Very important	93.0	91.5	95.0	
Somewhat important	6.6	7.9	4.7	
Not important	0.4	0.6	0.2	
In your opinion, which is the most important meal of the day for your child? (%)				0.009
Breakfast	80.6	77.7	84.9	
Lunch	17.6	20.1	13.8	
Dinner	1.8	2.2	1.3	

* Chi Squares tests of the proportions for significant difference between daily and non-daily breakfast intake.

**Table 7:** Multivariate analysis for selected anthropometric and lifestyle variables while controlling for age and socio-demographic factors stratified by gender and breakfast intake frequency. Data are means and standard deviations.

Variable	Gender	Breakfast Intakes		*p*-Value *
		<5 Days/Week (*N* = 685)	5 + Days/Week (*N* = 464)	
Body weight (kg)	Boys	33.5 ± 11.8	30.9 ± 9.6	Breakfast intake: 0.004
				Gender: 0.287
	Girls	33.8 ± 11.7	32.4 ± 11.3	Breakfast intake by gender interaction: 0.127
	All	33.7 ± 11.8	31.7 ± 10.5	
BMI (kg/m^2^)	Boys	18.2 ± 4.2	17.3 ± 3.6	Breakfast intake: 0.009
	Girls	18.4 ± 3.9	18.0 ± 3.9	Gender: 0.054
	All	18.3 ± 4.0	17.7 ± 3.8	Breakfast intake by gender interaction: 0.213
Screen time (h/night)	Boys	3.55 ± 1.8	3.04 ± 1.5	Breakfast intake: 0.081
				Gender: =<0.001=
	Girls	2.78 ± 1.5	2.81 ± 1.8	Breakfast intake by gender interaction: 0.005
	All	3.13 ± 1.7	2.91 ± 1.7	Breakfast intake: <0.001
Sleep duration (h/night)	Boys	8.18 ± 1.2	8.51 ± 1.1	Gender: 0.025
	Girls	8.30 ± 1.3	8.63 ± 1.0	Breakfast intake by gender interaction: 0.886
	All	8.25 ± 1.2	8.57 ± 1.1	
Physical activity (min/week)	Boys	196.0 ± 232.5	213.3 ± 225.5	Breakfast intake: 0.526
	Girls	95.2 ± 158.4	92.4 ± 157.8	Gender: <0.001
	All	141.2 ± 202.0	148.2 ± 201.1	Breakfast intake by gender interaction: 0.268

* Wilks’ Lambda *p* values: age <0.001; father age = 0.030; mother age = 0.048; father education = 0.002; mother education = 0.135; family income = 0.100; breakfast intake <0.001; gender <0.001 and breakfast intake by gender interaction = 0.056.

**Table 8:** Results of logistic regression analysis of selected lifestyle behaviors and overweight/obesity status relative to high versus low breakfast intake frequency among Saudi children, while adjusted for age, gender, and socio-demographic factors (*N* = 1138).

Variable	Breakfast Intake (<5 Days/Week Versus 5 + Days/Week) *			
	aOR	(95% CI)	SEE	*p*-Value
Age	1.008	0.934–1.088	0.039	0.837
Gender (girls = ref)	1.00			
Boys	1.026	0.79401.326	0.131	0.843
No. of family member in the house (high = ref)	1.00			
Low number	1.053	0.797–1.392	0.142	0.715
Paternal age (older age = ref)	1.00			
Younger age	0.937	0.774–1.135	0.098	0.507
Maternal age (older age = ref)	1.00			
younger age	0.953	0.755–1.202	0.119	0.684
Paternal education (high = ref)	1.00			
Low education	1.212	1.020–1.440	0.088	0.029
Maternal education (high = ref)	1.00			
Low education	1.212	1.003–1.464	0.096	0.046
Family income (> low = ref)	1.00			
High income	0.964	0.808–1.151	0.090	0.688
No. of children in the family (high = ref)	1.00			
Low number of children	0.848	0.673–1.069	0.118	0.163
Screen time (high = ref)	1.00			
Low screen time	1.026	0.784–1.344	0.138	0.849
Sleep duration (sufficient = ref)	1.00			
Insufficient sleep	0.735	0.567–0.951	0.132	0.019
Physical activity (active = ref)	1.00			
Inactive	0.899	0.636–1.271	0.177	0.548
Overweight or obesity (BMI ≥ 25 kg/m^2^ = ref)	1.00			
BMI < 25 kg/m ^2^	1.333	1.015–1.752	0.139	00.039

* Less than 5 days/week of breakfast intake was used as a reference category. aOR = adjusted odds ratio; CI = confidence interval; ref = reference category; SEE = standard error.
